# Modulation of protein behavior through light responses of TiO_2_ nanodots films

**DOI:** 10.1038/srep13354

**Published:** 2015-08-26

**Authors:** Kui Cheng, Yi Hong, Mengfei Yu, Jun Lin, Wenjian Weng, Huiming Wang

**Affiliations:** 1 School of Materials Science and Engineering, State Key Laboratory of Silicon Materials, Cyrus Tang Center for Sensor Materials and Applications, Zhejiang University, Hangzhou 310027, China; 2 The Affiliated Stomatologic Hospital, Zhejiang University, Hangzhou 310003, China; 3 The First Affiliated Hospital of Medical College, Zhejiang University, Hangzhou, 310003, China; 4 The Shanghai Institute of Ceramics, Chinese Academy of Sciences, 1295 Dingxi Road, Shanghai, 200050, China

## Abstract

In this work, the behavior of protein molecules adsorbed on TiO_2_ nanodots films are modulated through the light responses of the nanodots. TiO_2_ nanodots films are first prepared through phase separation induced self assembly. Then, bovine serum albumin (BSA) is adsorbed on TiO_2_ nanodots films and exposed to ultraviolet (365 nm) illumination. It is found the conformation of surface-bound BSA molecules changes with ultraviolet illumination. Moreover, the BSA molecules conjugate to the surface-bound molecules, which are in the overlayer, are released. The reason is ascribed to that TiO_2_ nanodots absorb ultraviolet and result in the increase of surface hydroxyl groups on nanodots. Such increase further leads to intensified attraction of -NH3 groups in the surface-bound BSA molecules. That not only changes the conformation of the surface-bound BSA molecules, but also weaken the conjugation between surface-bound molecules and other BSA molecules in the overlayer. Eventually, the overlayer of BSA molecules is released. It is believed that such protein conformation variation and release behavior induced through light responses of TiO_2_ nanodots are crucial in understanding the biomedical performance of TiO_2_ nanostructures. Also, it could be widely utilized in tailoring of the materials-protein interactions.

In the past decades, many works have been carried out on protein behavior on the surface of biomaterials. In fact, proteins begin to interact with biomaterials at the very beginning when in contact with biological environment^1^,^2^,^3^,^4^, and eventually determine the performance of biomaterials through influencing the responses of cells and the functions of other proteins^5^,^6^,^7^,^8^,^9^,^10^.

Protein adsorption on materials has been investigated by many researchers and several adsorption models of different proteins are proposed^11^,^12^,^13^,^14^. Though, few works look into the conformation and its evolution of adsorbed protein. In fact, the conformation change of protein molecules comes along with adsorption on solid surface^15^,^16^,^17^, and that not only influences the characteristics of adsorbed protein itself, but also directs the behaviors of surface. Effective control and optimization of protein conformation have been recognized as a hotspot in both protein and biomaterials researches. E. g., some researchers have reported that the applying of electric field may directly alter the conformation of the proteins through electrostatic attraction and repulsion of charged groups^18^,^19^.

In our previous study, a light induced cell sheet detachment phenomenon on TiO_2_ film was reported. Cell sheets with good livelihood could be obtained through simple ultraviolet exposure^20^. Also, it is found light responses of TiO_2_ bring excellent biological property through improved adsorption and up-regulated expression of certain proteins after light exposure^21^,^22^. Being photo responsible semiconductors, it is well known that TiO_2_ could easily convert photon energy to electron^23^,^24^,^25^, and then leads to varied surface charge. Therefore, these works may strongly imply that the light responses of TiO_2_ could effectively influence the behavior of proteins adsorbed.

In this work, we look into the behaviors of proteins adsorbed on TiO_2_ nanodots film with a multilayer adsorption model of bovine serum albumin (BSA). A light-induced conformation change of surface-bound protein and consequential protein release are demonstrated.

## Results and Discussions

A TiO_2_ nanodots (TN) film was used^26^. BSA was used as a model protein because serum albumin is the most abundant plasma protein in mammals and the main protein in the culture medium. Moreover, it is generally adsorbed in the first place^11^,^15^.

**Figure 1:**
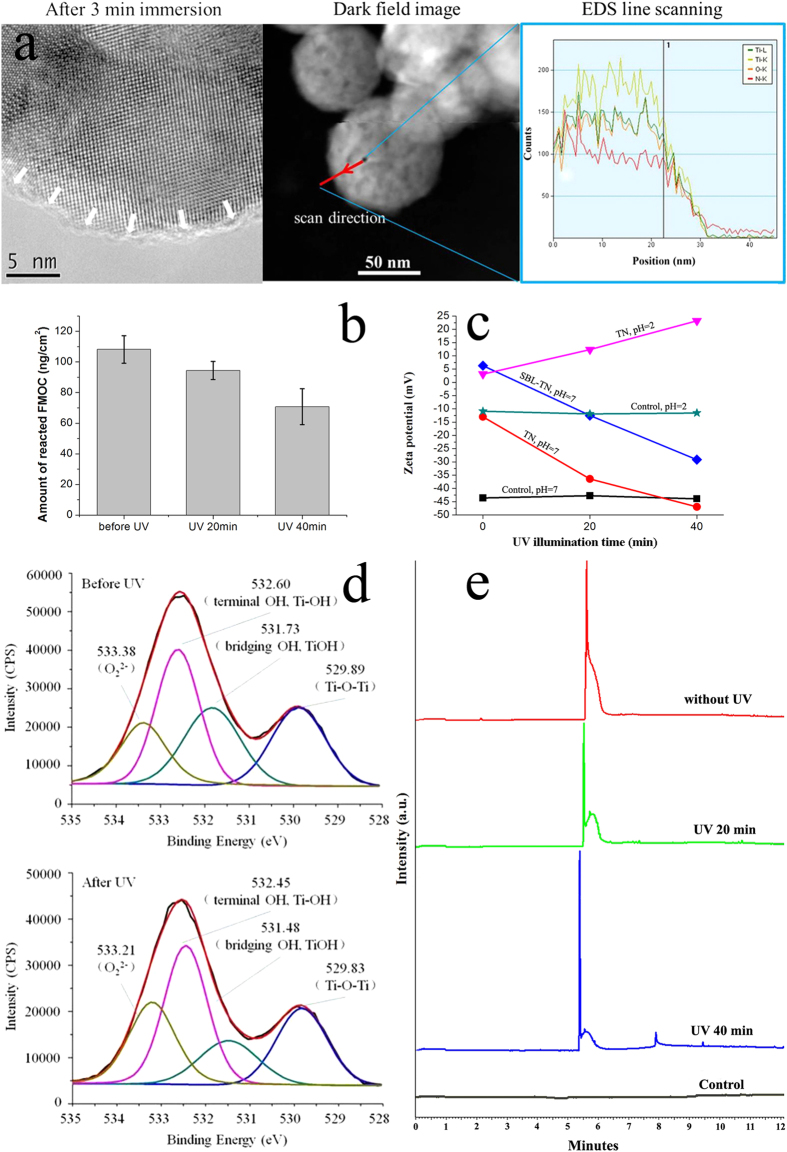
(**a**) TEM, EDS analyses of BSA adsorption on a typical individual TiO_2_ nanodot; (**b**) Amount of exposed -NH_3_ groups against different time of UV365 illumination; (**c**) Zeta potential of TiO_2_ nanodots against different time of UV365 illumination; (**d**) XPS results of TiO_2_ nanodots before and after UV365 illumination; (**e**) Capillary electrophoresis analyses of remained molecules after different time of UV365 illumination.

Transmission electron microscopy (TEM) was used to observe protein adsorbed on a typical individual TiO_2_ nanodot. As shown in Fig. 1a, a 1 ~ 2 nm adsorption layer was observed on the TiO_2_ nanodot after 3 min immersion in BSA solution. The energy dispersive spectroscopy (EDS) line scanning result showed that nitrogen signal decreased gradually from the center to the edge on the TiO_2_ nanodot. Obviously, a thin BSA layer could be easily adsorbed on individual nanodots. This layer could be regarded to be bound to TiO_2_ directly (surface-bound layer). Also, since TiO_2_ nanodots film consists of numerous similar nanodots, it is considered that macroscopically a BSA layer with similar thickness was adsorbed on the nanodots film.

**Figure 2:**
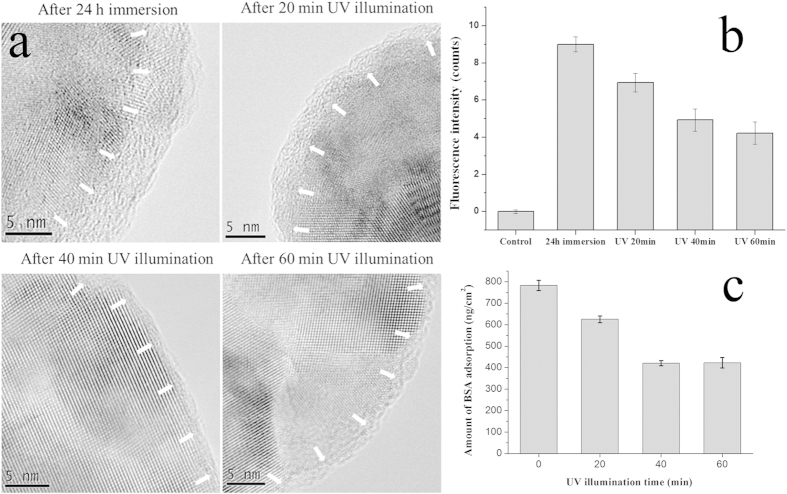
(**a**) TEM micrographs of BSA adsorption thickness evolution against different time of UV365 illumination; (**b**) Fluorescence intensity variations of CLSM photos against different time of UV365 illumination; (**c**) QCM quantitative results on BSA desorption against different time of UV365 illumination.

In order to evaluate the status and evolution of the BSA adsorbed, a standard method based on 9-fluorenyl-methyl chloroformate (FMOC)^27^,^28^,^29^ is adopted to evaluate the amount of exposed -NH_3_^+^ in this BSA layer. Briefly, FMOC reacts with exposed -NH_3_^+^ and remains on the surface, and the mass increment is proportional to the amount of -NH_3_^+^ exposed. In Fig. 2b, before UV365 illumination, the mass increase is 108.2 ng/cm^2^; it drops to 94.4 ng/cm^2^ after 20 min UV365 illumination, and then 70.8 ng/cm^2^ after 40 min. That is to say, 40 min UV365 illumination leads to about 34.6% decrease of exposed -NH_3_^+^. More information was given by the surface zeta potential variations of the BSA adsorbed TiO_2_ nanodots film, as shown in Fig. 1c (BSAMO-TN). Before UV365 illumination, the zeta potential of the surface is positive, which means positive groups, most probably -NH_3_^+^, are exposed; while the zeta potential turns negative with increasing UV365 illumination time. Such transition of zeta potential actually implies that the exposed positively charged groups decrease with increasing UV illumination time. That is in well agreement with the result of -NH_3_^+^ reduction obtained with FMOC method in Fig. 1b.

What happens to these molecules during UV365 illumination? In order to elucidate that, the zeta potential of TiO_2_ nanodots films under different pH value and UV365 illumination time were also measured and shown in Fig. 1c. Obviously, the surface zeta potential of TiO_2_ nanodots film decreased with UV365 illumination at pH 7, which is exactly the same trend to BSA adsorbed one. Also, no zeta potential change was observed for the control nanodots free silica substrate. In fact, it is reported that the reactive surface hydroxyl groups of TiO_2_ deprotonate or protonate when the environmental pH value is higher or lower than the isoelectric point^30^. Therefore, it is reasonable to ascribe such negative shift against UV365 illumination time to the generation and deprotonation of light induced reactive surface hydroxyl groups. The positive shift of zeta potential against UV365 illumination time at pH 2, which is lower than the isoelectric point, also proved that there do be more reactive surface hydroxyl groups under UV365 illumination. X-ray photoelectron spectroscopy results in Fig. 1d further prove the increase of reactive surface hydroxyl groups: it was found more terminal -OH groups^12^,^31^, which are reported to be more reactive, were observed on the post-illumination sample. In supplementary information Figure S1, Kelvin probe force microscope also proved the surface potential responded to UV365 exposure. Obviously, these potential variations definitely affected the state of BSA molecules adsorbed.

In order to clarify how the adsorbed BSA molecules were affected, the adsorbed BSA layer before and after UV365 illumination were eluted through a SDS method and characterized with capillary electrophoresis, as shown in Fig. 1e. In comparison with untreated BSA, the eluted BSA molecules showed almost the same molecular weight and no obvious signal of small molecule was detected. That actually means BSA molecule remained its integrity during UV365 illumination. That is to say, based on the above results, the decrease of exposed -NH_3_^+^ through UV365 illumination actually means UV365 illumination could effectively modulate the conformation of BSA molecules through the responses of TiO_2_ nanodots films.

In many cases, protein adsorbed onto solid surfaces in multilayers. Whether the above mentioned light response of TiO_2_ nanodots films could influence the protein molecules beyond the surface-bound layer is another interesting question. As shown in Fig. 2a, after soaking in BSA solutions for 24 h, a much thicker BSA layer is adsorbed. With increasing time of UV365 illumination, decreasingly thin BSA layers were clearly observed on individual TiO_2_ nanodots for the initial 40 min, and then the thickness of the layer kept almost unchanged at elongated illumination time. The thickness of the residual layer is about 1 ~ 2 nm, which is almost the same to that in Fig. 1a. For the TiO_2_ nanodots film case, in Fig. 2b, macroscopically, the fluorescence intensity of adsorbed BSA also decreased gradually with increasing UV365 illumination time, and kept almost unchanged after 40 min (refer to supplementary information Figure S2 for original photos). Such result means that extensively protein release through UV365 illumination occurred on numerous TiO_2_ nanodots. However, some BSA molecules kept adsorbed on the surface of TiO_2_ nanodots steadily under UV365 illumination.

Quantitative results of protein release were obtained through quartz crystal microbalance (QCM) characterization. TiO_2_ nanodots were immobilized on the crystal surface of quartz crystal microbalance (QCM), and then exactly the same soaking and UV365 illumination process were carried out. It was found with UV365 illumination, as shown in Fig. 2c, a continuous mass loss up to 47.0% was observed within the first 40 min; while the rest kept adsorbed even after 60 min. In comparison, no such mass loss was observed on those TiO_2_ nanodots films immersed in BSA solution for 3-min (supplementary information Figure S3). That means such protein molecules release only happens when multilayer adsorption occurs.

**Figure 3:**
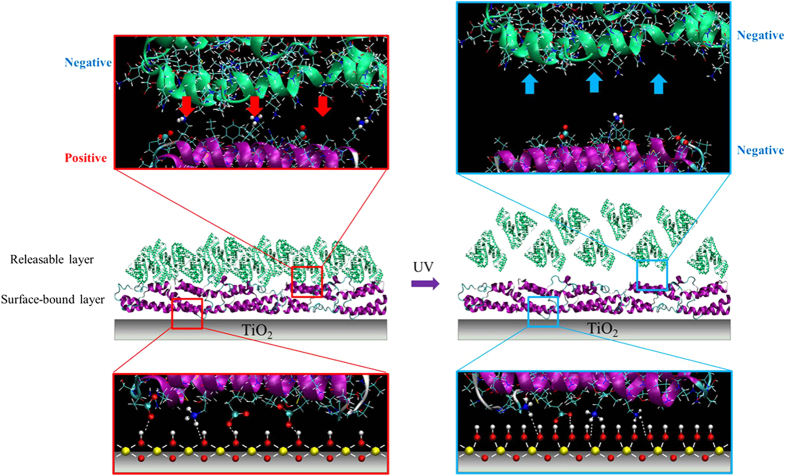
Diagram of UV365 illumination on the behaviors of BSA adsorbed on TiO_2_ nanodots film.

Clearly, Fig. 2 indicates that the light responses of TiO_2_ nanodots films not only leads to the conformation variation of surface-bound BSA layer, but also eventually affect the behavior of the exterior adsorption layer of BSA molecules. The diagram of whole process was presented in Fig. 3. UV illumination induces more -OH on TiO_2_ nanodots surface; such variation enhances the electrostatic attraction and repulsion with correspondent groups in the adsorbed protein molecules and then induces conformational changes of these molecules in the surface-bound layer. These changes subsequently change the conjugation between the exterior layer and the surface-bound layer of protein, and eventually lead to the release of the exterior layer of protein.

Many works have reported that ultraviolet illumination on nano TiO_2_ could lead to the formation of oxygen vacancies, and then release free radicals to kill bacteria and decompose organic molecules. In this work, it is actually found that through careful selection of wavelength and illumination time, ultraviolet illumination on nano TiO_2_ could show unusual effects on protein molecules adsorbed: light induced hydroxyl groups lead the surface bound protein molecules to change their conformations, and eventually result in more closely attached surface bound protein molecules and gradual release or detach of the molecules in the outer layer. Such finding serves well for many biological properties of TiO_2_. E.g., many works have reported that ultraviolet illuminated TiO_2_ showed improved cellular responses and attributed that to the improved protein expression^21^,^32^. Our work further support that such improvement may actually originates from the protein molecules adsorbed on ultraviolet illuminated TiO_2_ in different conformations.

More importantly, it also demonstates that proper ultraviolet illumination could be utilized as an effective way to directly modulate the behavior of protein molecules even after they are adsorbed on nano TiO_2_ based materials. As a result, the interactions between the corresponding materials and cells could be effectively tailored or improved. That provides a feasible approach to improve the performance of bioimplants, or even imparts them with additional functions. E.g., in supplementary information Figure S4, the potential light controlled release property of TiO_2_ nanodots film with surface-bound BSA was shown. A collagen layer was adsorbed, and then released after UV365 illumination. That actually means other functional protein or peptide molecules could be effectively loaded and released through light responses of TiO_2_ based materials. Moreover, it is expected that such adsorption and release behavior controlled through light response of materials could be potentially applied in many areas, such as surface functionallization of implants, controlled release of drugs, protein purification and etc.

## Conclusion

In conclusion, it is observed that the light responses of TiO_2_ could be utilized to modulate the behaviors of adsorbed protein molecules. Upon UV365 illumination, TiO_2_ nanodots absorb ultraviolet and lead to increase of surface hydroxyl groups, such increase attracts more -NH_3_^+^ groups from surface-bound BSA molecules and results in conformation changes. The conformation changes weaken the conjugation between surface-bound molecules and other molecules. As a result, the overlayer of BSA molecules are released from the surface of TiO_2_ nanodots through UV365 illumination. Such modulation shows much potential in many research areas related to materials-protein interactions.

## Methods

### Preparation of TiO_2_ nanodots film

TiO_2_ nanodots films (TN) were prepared on the substrate through a phase separation-induced self-assembly method^26^. Briefly, a precursor sol containing titanium tetrabutoxide (Sinopharm Chemical Reagent, CP, >98%), acetylacetone (Lingfeng Chemical Reagent, AR, >99%) and polyvinyl pyrrolidone (K30, Sinopharm Chemical Reagent, AR, >99%), was spin-coated on the quartz or quartz crystal microbalance (QCM) crystal surface and allowed to phase-separation. After further heat treatment at 500 °C. A TiO_2_ nanodots film was obtained. The diameter of the nanodots ranged from 30 to 110 nm, and the dot density was about 5.6 × 10^10^/cm^2^. The dots were polycrystalline.

### BSA adsorption

BSA was dissolved in phosphate-buffered saline (PBS, 0.01 M, pH 7.2) to form 1% (wt/vol) solution. For fluorescence assay, Sulfo-NHS-LC-Biotin (Pierce Biotechnology, 21335) was conjugated to BSA. Excess volume of 10 mM biotin reagent solution was added to the BSA solution. After incubation in ice for two hours, the labeled protein was purified using desalting to remove non-reacted and hydrolyzed biotin reagent.

For multilayer adsorption, excess BSA solution was dripped on TN-coated quartz or QCM crystal and stored in an oven held at 25 °C 24 h, then the quartz or crystal were washed with deionized water for 3 times; while only 3 min oven storage was taken for monolayer adsorption case. For fluorescence assay, TN-coated quartz was immersed in excess biotinylated BSA for 24 h at 25 °C, then washed by deionized water for 3 times.

### Transmission electron microscopy

All of the samples were observed by TEM (FEI, F-20) at an accelerating voltage of 200 kV. For 24 h BSA solution immersed samples, the samples were washed by deionized water for 3 times, then, after certain time ultraviolet (UV, 365 nm) illumination, the BSA adsorbed TNs were scraped from substrates. For 3 min BSA solution immersed samples, the samples were washed by deionized water for 3 times, and then scrape off directly.

### UV365 resources and illumination method

A cold LED UV light was used to eliminate any interference by heat. A UV light with a wavelength of 365 nm and a power of 2.0 mW/cm^2^ was used. The transmittance power was measured to be 1.4 mW/cm^2^. As calculated following the longest illumination time in this work (40 min), the total energy was about 3360 mJ/cm^2^, much lower than the reported safe value (7500 mJ/cm^2^).

### Preparation of 9-Fluorenylmethyl succinimidyl carbonate for amino group detection

9-Fluorenylmethyl succinimidyl carbonate (FMOC-OSu, sigma) was dissolved in acetonitrile to form 1% (wt/vol) FMOC-OSu solution; the pH was adjusted to 8.5 by sodium tetraborate^33^.

### Detecting the amount of protein adsorptions and reacted FMOC

The quartz crystal microbalance with dissipation technique (QCM-D) (E4 Q-Sense, Biolinscientific, Sweden) was used to detect mass changes of adsorbed BSA and reacted FMOC before and after UV illumination. Gold coated quartz crystals (5 MHz) were used as substrates. TN was prepared on the crystals through the method mentioned above. The mass changes on the crystal were calculated using the Sauerbrey equation:


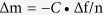


C = 17.7 ng Hz^−1^ 1^ ^cm^−2^ for a 5 MHz quartz crystal; Δf is the changes in the QCM steady state frequency after adsorption; n = 1, 3, 5, 7 and is the overtone number; Each sample was washed thoroughly in deionized water for 3 times, dried under nitrogen stream, and then measured.

### Confocal laser scanning microscopy

After immersing in excess biotinylated BSA for 24 h at 25 °C, TN-coated quartz samples were illuminated with UV365 for 20, 40 and 60 minutes respectively. Then the samples were washed by deionized water for 3 times and dried. After that the samples were used for directly observation with confocal laser scanning microscopy (CLSM, Olympus, IX81). A TN-coated quartz without BSA solution immerion was also observed as a control. The fluorescence intensity values of CLSM photos were plotted against UV365 illumination time to indicate the amount of BSA adsorbed.

### Zeta-potential measurement

The zeta-potential measurement was performed by using a particle size and zeta-potential analyzer (Delsa Nano C, Beckman-Coulter). The samples were measured in 0.9% NaCl solution. The pH was adjusted by the addition of HCl or NaOH solution. The results presented are average of five independent measurements. TN-free quartz (1 × 2 cm^2^) was used as a control. Before the measurement, all samples were immersed in sample cells with pH adjusted NaCl solution for 10 min to stabilize the electric double layer.

### Surface hydroxyl groups

X-ray photoelectron spectroscopy (XPS, Kratos AXIS Ultra DLD) was used to characterize the surface composition of TiO_2_ nanodots before and after UV365 illumination with an Al Kα source (1486.6 eV). A detailed scan for O and Ti was carried out with a step of 0.1 eV. The Ti 2p peak (458.0 eV) was used for calibration.

### Detecting BSA molecule status by capillary electrophoresis

The adsorbed BSA on unilluminated, 20-min and 40-min UV365 illuminated TN was washed off with 1 wt% sodium dodecyl sulfonate (SDS). A Beckman P/ACE 5000 capillary electrophoresis system (Beckman Coulter Instrument, Fullerton, CA, USA) was preconditioned by successively flushing with 0.1 mol/L NaOH for 20 min, H_2_O for 5 min and running buffer for 5 min. Before each run, the capillary was flushed consecutively with 0.1 mol/L NaOH, H_2_O and running buffer for 1 min. Hydrodynamic injection was carried out under N_2_ pressure at 0.5 psi for 10 seconds. Detection wavelength was set at 214 nm. All electrophoresis runs were performed at a temperature of 25 °C^34^. A bare fused-silica capillary (Capillary 1 from Xinnuo Fiber Sepu, China) with 100 um i.d. and effective length of 20 cm was used. The separations were performed at a constant voltage of 10 kV (current of about 40 μA). The running buffer contained 100 mmol/L H_3_BO_3_, 10 mmol/L SDS and 2 g/L HEC at pH 8.8. The sample solution consists of 15 mmol/L H_3_BO_3_ and 20 mmol/L SDS at pH 8.6. All the data was collected and analyzed using the P/ACE Station software.

## Additional Information

**How to cite this article**: Cheng, K. *et al.* Modulation of protein behavior through light responses of TiO2 nanodots films. *Sci. Rep.* **5**, 13354; doi: 10.1038/srep13354 (2015).

## Supplementary Material

Supplementary Information
