# Supplementary material for: Modulation of protein behavior through light responses of TiO2 nanodots films
Source: Sci Rep. 2015 Aug 26;5:13354. doi: 10.1038/srep13354 (PMC4549798; doi:10.1038/srep13354)
Supplement: Supplementary Information [file srep13354-s1.pdf]

Supplementary Information

Modulation of protein behavior through light responses of TiO_2 nanodots films

Kui Cheng, Yi Hong, Mengfei Yu, Jun Lin, Wenjian Weng[*]*, Huiming Wang*[*]

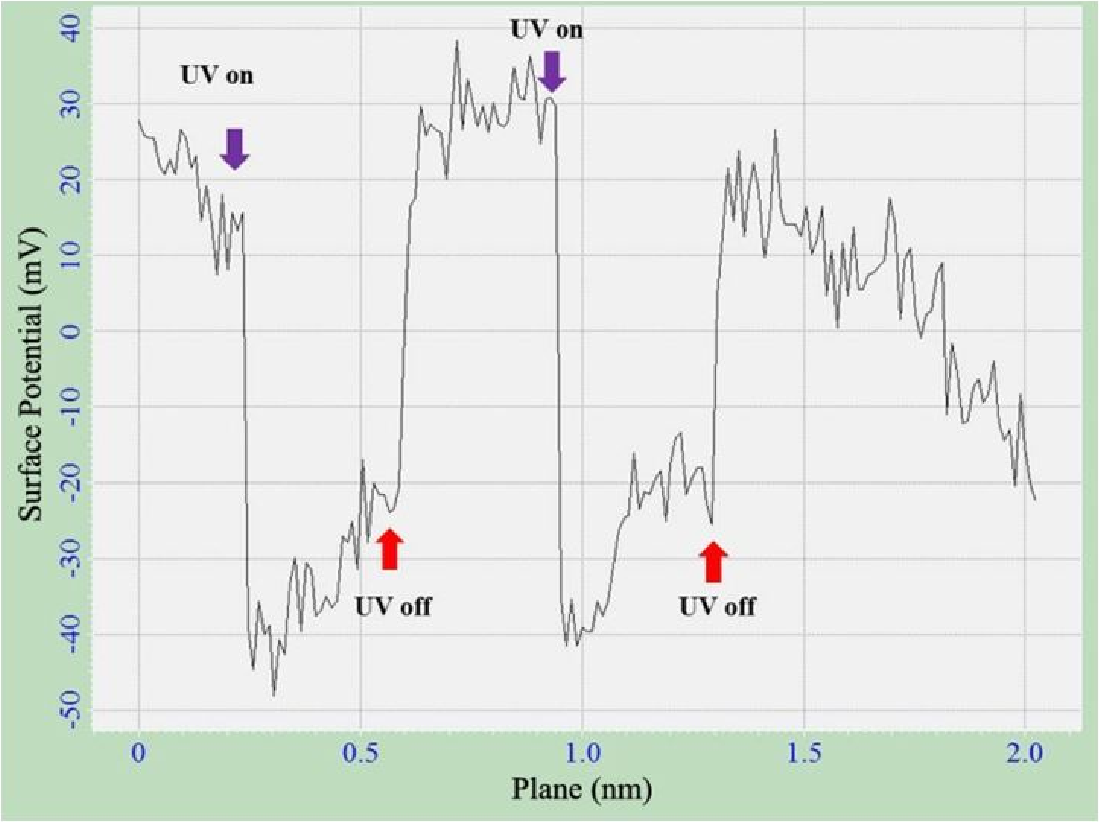

Figure S1 Kelvin probe force microscope analysis of TiO_2 nanodots film under UV365 illumination.

Method: TiO_2 nanodots film was directly with a Kelvin probe force microscope in ambient environment.

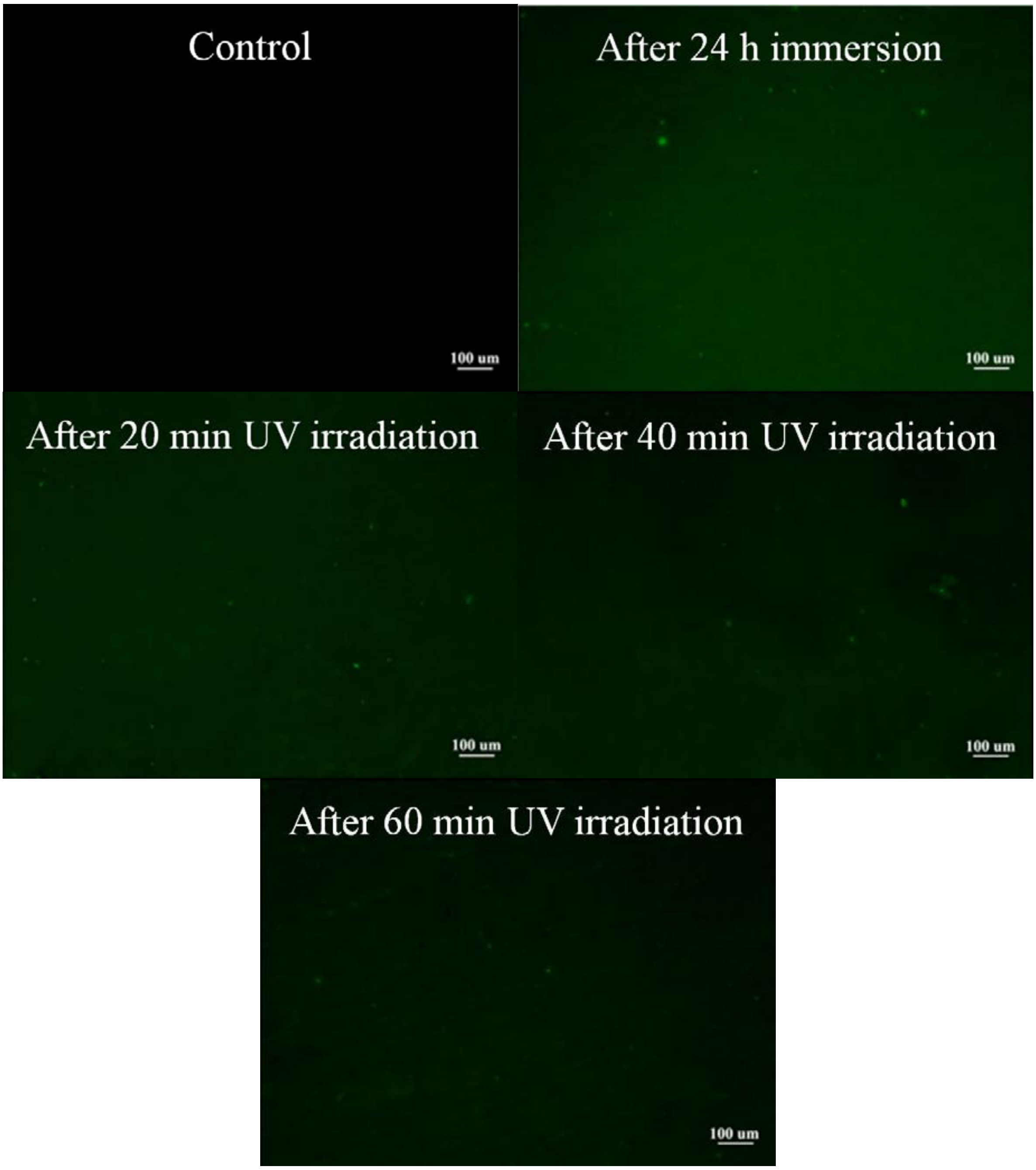

Figure S2 CLSM photos of BSA adsobed TiO_2 nanodots films after different time of UV365 illumination

Method: As mentioned in “*Confocal laser scanning microscopy*” section in experimental part.

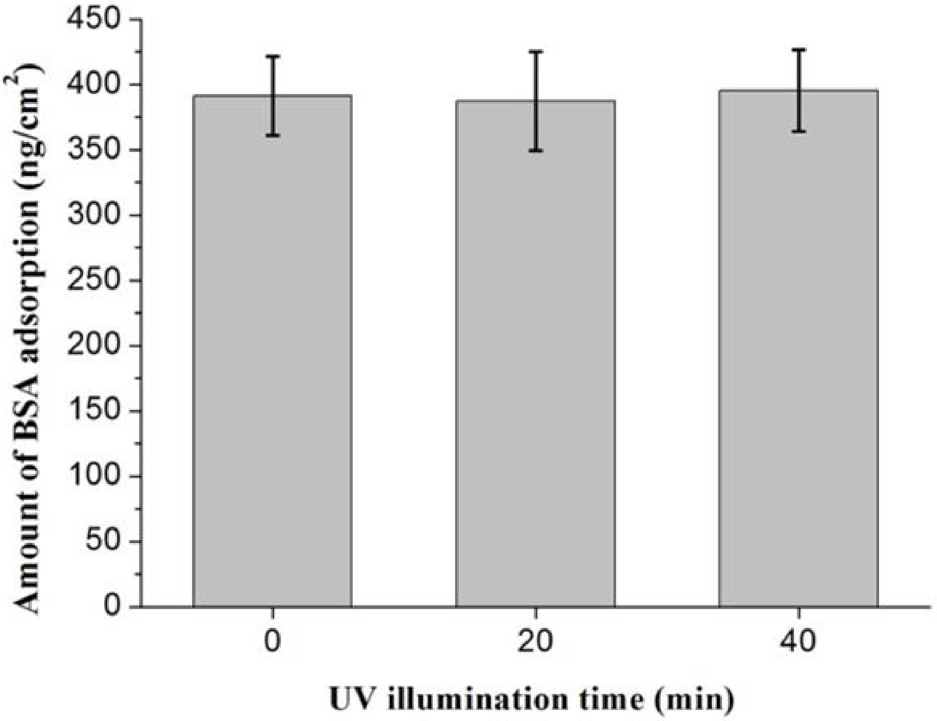

Figure S3 QCM quantitative results on surface-bound BSA adsorption against different time of UV365 illumination

Method: As mentioned in "*Detecting the amount of protein adsorptions and reacted FMOC*" section in experimental part.

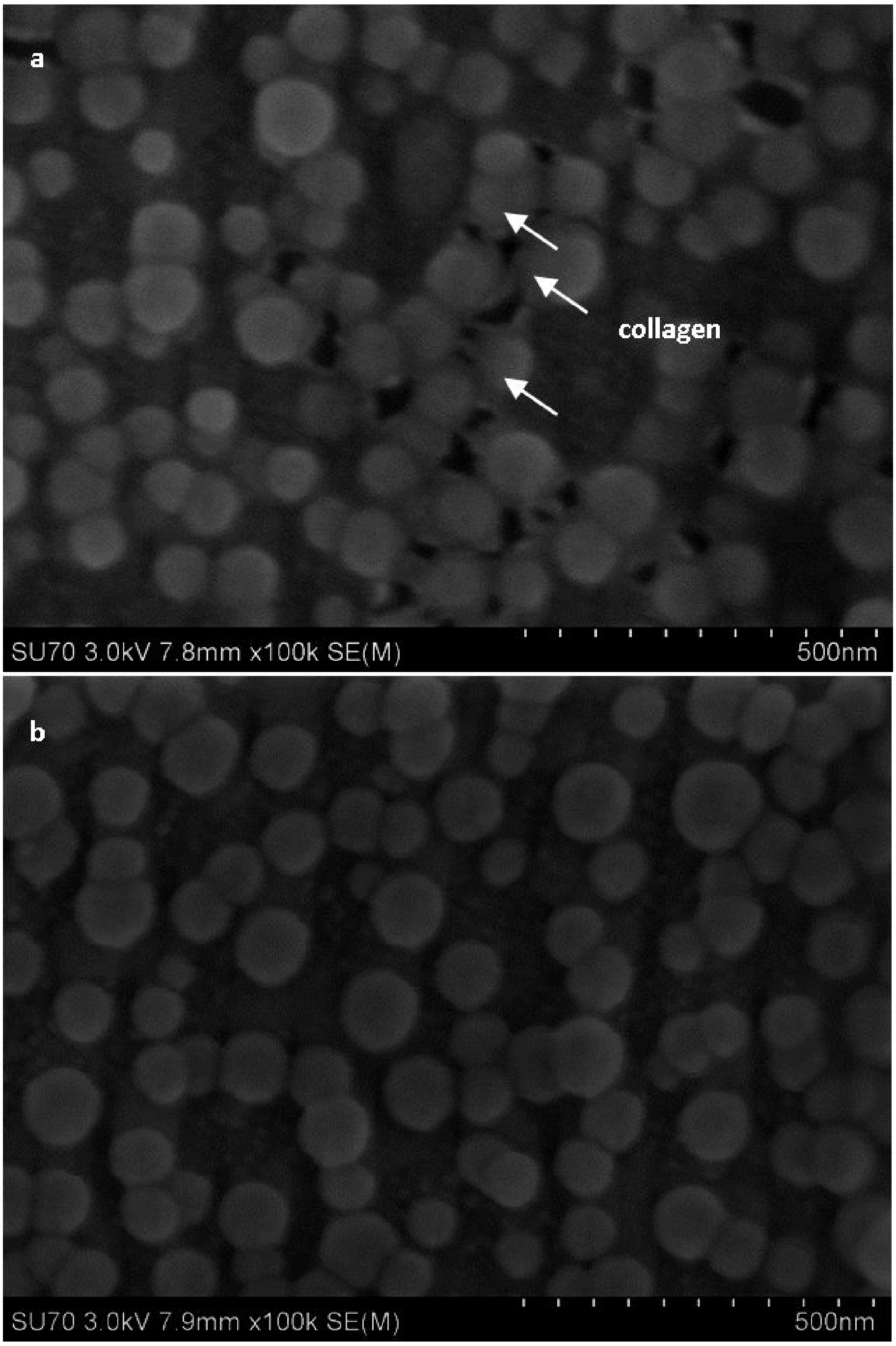

Figure S4 Morphology of collagen loaded TiO_2 nanodots films with surface-bound BSA (a) before and (b) after UV365 illumination

Method: TiO_2 nanodots films were placed in 24 well cell culture plate, then 0.5 ml aqueous solution of type I collagen with a concentration of 0.5 mg/ml was added and allowed for 24 hours. After that, TiO_2 nanodots film was transferred into phosphate buffered solution and illuminated with UV365 for 20 min. Morphology of the films before and after UV365 illumination were observed with a scanning electron microscope (SEM, FEI, SU-70).
